# Supplementary material for: Surgeon- and hospital-level variation in wait times for scheduled non-urgent surgery in Ontario, Canada: A cross-sectional population-based study
Source: PLoS One. 2024 Aug 8;19(8):e0307845. doi: 10.1371/journal.pone.0307845 (PMC11309399; doi:10.1371/journal.pone.0307845)
Supplement: Attachment — Submitted filename: Response to reviewer comments 19Jun24.docx [file pone.0307845.s001.docx]

Dr. Gianni Virgili

Academic Editor

PLOS ONE

19 June 2024

Dear Dr. Gianni Virgili

Thank you for reviewing our manuscript, “Surgeon- and hospital-level variation in wait times for scheduled non-urgent surgery in Ontario, Canada: A cross-sectional population-based study” (REF: PONE-D-24-09785) for publication in PLOS ONE.

We value the reviewer’s considered and thoughtful comments. Included in this resubmission is a:

- Revised manuscript with all changes highlighted in yellow
- Revised manuscript without changes highlighted
- Response to each of the reviewer comments

We look forward to your response.

Sincerely,

Pieter de Jager

| Comment | Response |
| --- | --- |
| General comments: | |
| Please ensure that your manuscript meets PLOS ONE's style requirements, including those for file naming. |  |
| Please provide additional details regarding participant consent. In the ethics statement in the Methods and online submission information, please ensure that you have specified (1) whether consent was informed and (2) what type you obtained (for instance, written or verbal, and if verbal, how it was documented and witnessed). If your study included minors, state whether you obtained consent from parents or guardians. If the need for consent was waived by the ethics committee, please include this information. If you are reporting a retrospective study of medical records or archived samples, please ensure that you have discussed whether all data were fully anonymized before you accessed them and/or whether the IRB or ethics committee waived the requirement for informed consent. If patients provided informed written consent to have data from their medical records used in research, please include this information. | We added these sentences to this section:  The data were fully anonymized, linked using unique encoded identifiers, and analyzed at ICES.  ICES is an independent, non-profit research institute whose legal status under Ontario’s health information privacy law allows it to collect and analyze health care and demographic data, without consent, for health system evaluation and improvement.  The use of the data in this project is authorized under section 45 of Ontario’s Personal Health Information Protection Act (PHIPA) and does not require review by a Research Ethics Board. |
| Thank you for stating the following financial disclosure:  "This project was funded by the Canadian Institutes of Health Research through a project grant (no. PJT-166108)."    Please state what role the funders took in the study.  If the funders had no role, please state: ""The funders had no role in study design, data collection and analysis, decision to publish, or preparation of the manuscript.""  If this statement is not correct you must amend it as needed.  Please include this amended Role of Funder statement in your cover letter; we will change the online submission form on your behalf. | We’ve included the following statement in the Ethics section: This project was funded by the Canadian Institutes of Health Research through a project grant (no. PJT-166108). The funders had no role in study design, data collection and analysis, decision to publish, or preparation of the manuscript. |
| We note that your Data Availability Statement is currently as follows: All relevant data are within the manuscript and its Supporting Information files. Please confirm at this time whether or not your submission contains all raw data required to replicate the results of your study. Authors must share the “minimal data set” for their submission. PLOS defines the minimal data set to consist of the data required to replicate all study findings reported in the article, as well as related metadata and [methods](https://journals.plos.org/plosone/s/data-availability#loc-minimal-data-set-definition). For example, authors should submit the following data:  The values behind the means, standard deviations and other measures reported; The values used to build graphs; The points extracted from images for analysis.  Authors do not need to submit their entire data set if only a portion of the data was used in the reported study. If your submission does not contain these data, please either upload them as Supporting Information files or deposit them to a stable, public repository and provide us with the relevant URLs, DOIs, or accession numbers. For a list of recommended repositories, please [see](https://journals.plos.org/plosone/s/recommended-repositories) . If there are ethical or legal restrictions on sharing a de-identified data set, please explain them in detail (e.g., data contain potentially sensitive information, data are owned by a third-party organization, etc.) and who has imposed them (e.g., an ethics committee). Please also provide contact information for a data access committee, ethics committee, or other institutional body to which data requests may be sent. If data are owned by a third party, please indicate how others may request data access. | The raw dataset from this study is held securely in coded form at ICES. While legal data sharing agreements between ICES and data providers (e.g., healthcare organizations and government) prohibit ICES from making the dataset publicly available, access may be granted to those who meet pre-specified criteria for confidential access, available at www.ices.on.ca/DAS (email: [das@ices.on.ca](mailto:das@ices.on.ca)).  The full dataset creation plan and underlying analytic code are available from the authors upon request, understanding that the computer programs may rely upon coding templates or macros that are unique to ICES and are therefore either inaccessible or may require modification. |
| Please ensure that you include a title page within your main document. You should list all authors and all affiliations as per our author instructions and clearly indicate the corresponding author. | Added |
| Please include captions for your Supporting Information files at the end of your manuscript, and update any in-text citations to match accordingly. Please see our Supporting Information guidelines for more [information](http://journals.plos.org/plosone/s/supporting-information.): | We do not have any supporting files |
| METHODS & STATISTICAL COMMENTS: | |
| This is an interesting study trying to measure waiting list time variation at the hospital and surgeon level in Ontario.  One reviewer has concerns on statistical analyses. The normalizing transformation of the response variable (wait 2) is reported to normalize the residuals; in fact, the assumption in linear regression is homoskedasticity (similar residuals across covariate levels), rather than the normality of the response variable.  I suggest the authors are clearer regarding what covariates were planned in the protocol as the fixed part of the model, with changes to the protocol explained. Covariates could also be used in exploratory analyses of how the VPC/ICC is reduced once important covariates are introduced one at a time, e.g. age, sex, deprivation index, fragility indexes if available, etc. The authors could also consider whether surgical volume could be introduced as a random slope at the surgeon level: this would allow to test whether low vs high volume surgeons have different wait 2 time. | A squared logarithmic transformation with the most extreme 1% of wait times excluded resulted in an approximately normal distribution, with residual plots showing no sign of heteroscedasticity (now noted on Line 145).  The regression models were fitted using the *mixed* procedure (proc mixed, SAS/STAT version 15.3) and the method of restricted (or residual) maximum likelihood. The primary assumption of this method is that the data are normally distributed.  SAS Institute Inc. 2018. *SAS/STAT® 15.1 User’s Guide*. Cary, NC, Chapter 81 The MIXED Procedure.  The analysis plan, which effectively ignored fixed effects covariates such as age, sex, marginalization, as well as surgical volume, was intentional and defined a priori.  We had included the statement “Since the analysis aimed to quantify the observed variation in wait time due to surgeons and hospitals, we did not include explanatory fixed effects in the models” in the methods in anticipation of this concern.  We added the following sentences to the discussion to clarify the rationale behind this approach, and explicitly identify it as a limitation:  The protocol for this study was defined a priori to exclude fixed effects covariates such as age, sex, marginalization, as well as surgical volume. Our intent was not to explain any of the reasons behind the observed variation in wait times. Rather, our purpose was to describe the extent of this variation, with the intent of stimulating the development and implementation of health policy interventions that can attenuate this effect. The explanations for the variations in wait times we observed are important and should be the subject of future research. |
| The paper aimed to quantify the observed variation in wait time due to surgeons and hospitals. Overall, the paper is well-written and structured and used adequate statistical approach to estimate the variance partition coefficient (VPC) for the surgeon and the hospital effects. There are some concerns that could improve the paper if addressed:  The Wald test was applied to test the hypothesis H0: VPC=0 vs H1: VPC>0. As the VPC only nonnegative variances are allowed, there is a boundary (i.e., 0) in the variances’ parameter space, and regular inference statistical procedures for such a parameter could be problematic. | We thank the reviewer for pointing out the potential shortcomings of the Wald test in this application.  In place of Wald tests, we conducted a series of Likelihood Ratio tests which compared the two-intercept model to models having one of each intercept (physician level only and hospital level only), and each of those one-intercept models to a null model with no random effects. This is now noted on Lines 156-158.  For all procedures, all 4 tests showed the variances were highly significant (p < 0.00001).  Reference: Austin, P., Leckie, G. The effect of number of clusters and cluster size on statistical power and Type I error rates when testing random effects variance components in multilevel linear and logistic regression models. JOURNAL OF STATISTICAL COMPUTATION AND SIMULATION  2018, VOL. 88, NO. 16, 3151–3163. |
| Although the authors mentioned that fixed effects were not considered, it would be interesting to conduct an analysis incorporating fixed effects, provided such data are available. Are there any independent factors (surgeons/hospitals) associated with a longer time to surgery? | We agree with this comment and intend to investigate this in future research. As we explain in our response above, these associations were beyond the scope of this study. The additions to the discussion section on limitations should explain this better to readers. |
| What was the significance level value? | Added to Statistical Analysis section: We used a significance level of 0.05 to denote statistical significance. |
| Which statistical software was utilized in the data analysis? | Added to Statistical Analysis section: Analyses were performed using SAS 9.4. |
| I’m going to recommend this study be reviewed by a statistician. My concerns relate to the transformation of the outcome to make the distribution normal. Issues with this is that back-transformation is not always straight forward (smearing factors have been shown to have their own problems), and GLMs with different link functions and distributional families may be well-suited for skewed data, even with zeros. By transforming the outcome, the variability in the outcome is affected (reduced), so if you’re measuring variability, then you’ve effectively reduced the variability in variability, affecting the statistical analysis. | Without fixed effects in the model, no back-transformation was required to quantify the association between outcome and fixed effects. Since we report the variation in the transformed variable and conduct all the analyses with the transformed variable, the concern highlighted by the reviewer would not lead us to underestimate the true variation in outcome. |
| Where was the study data analyzed? E.g. ICES vs OH vs WC | Added to Statistical Analysis section: The data were linked using unique encoded identifiers and analyzed at ICES. |
| I agree that the especially long-waiters attract the most attention. So I wonder if those >99th percentile are truly outliers (data entry errors) or truly long-waiters that should have been retained in the analysis. Thoughts on this? With the flexibility of GLMs, transformation of the outcome may not be required, so this reason for omission may be moot. | These observations were excluded out of the necessity to avoid violating the model assumptions.  We assessed the use of a gamma model (with and without a log link function) for the outcome and found the untransformed wait times were not consistent with that distribution, with or without the top 1% of wait times.  We revised this potential limitation in the discussion:  Third, we excluded procedures with wait times at the extremes to allow transformation to a normal distribution; this might have biased our analysis towards underestimating the extent of variation. |
| In the section on data sources, please provide additional information on the WTIS. What kind of coverage does the WTIS have? All patients? All procedures? All hospitals (e.g. this excludes specialty hospitals like Shouldice, but I’m not sure about others). This may be particularly important for cataract surgeries if done at private facilities. | The WTIS reports on 59 surgical wait time data elements from more than 3,300 surgeons across 132 healthcare sites in Ontario. For the procedures we studied, including cataract surgery, this covers the vast majority of these procedures in Ontario. |
| After excluding procedures occurring within 1 year of a previous surgery of the same type, is this expected to remove the second stage of a staged procedure? Please make that explicit | Correct: Surgery on potentially bilateral sites, such as arthroplasty, could be intentionally staged, skewing the subsequent procedures' wait times and undermining the assumption of independence (included explicitly in “Outcome and Exposures”) |
| Please reference Table 1 in the methods section where the exclusions are described. What is the difference between a wait-2 of 0 days (excluded from the study) versus 1 day (included in the study)? Does it not still suggest data validity questions for non-urgent cases? | Table 1 has been moved to and referenced in the methods section  Given that these were all elective cases, it is extremely unlikely that a patient would be seen by a surgeon and receive their surgery on the same day – elective lists are booked in advance and patients are required to be fasted before coming for surgery. It is however conceivable that a new surgeon, or low volume surgeon could have a ‘next day’ opening on their elective list and book a patient with sort notice. The main reason for excluding records with wait time of 0 is they are implausible and indicative of data entry errors, e.g., the decision to treat date was entered into the field for procedure date, or vice-versa. There are plausible explanations for short waits of 1 day or more, e.g., openings in schedule created by cancellation of other procedures. Further, wait times of 0 days were excluded as a means to avoid violating the model assumptions.  We added this sentence to “Outcomes and Exposures”: Further, we excluded these observations to avoid undermining modelling assumptions. |
| RESULTS: | |
| Please elaborate on the sentence “If every surgeon had the same mean wait time, the VPC for the surgeon estimate would be 0”. As I understood from the methods, this is a measure of variability, not central tendency. Physicians can have the same mean wait-time, but vastly different distributions in wait-times. I think this is partly explained by the next sentence, but some clarity would be helpful. | Thank you, we have rephrased this to ‘if every surgeon had the same wait time distribution’ |
| Figure 2 x-axis: modify the labels to every 10 or so to make it readable. | We have deleted the labels, since they are not meaningful, and the vertical bars simply represent the ordered data according to provider unit. |
| DISCUSSION & CONCLUSION | |
| The authors provide a well-written manuscript on surgeon- and hospital-level variation in the time from decision-to-treat until surgery for the top non-urgent procedures in Ontario, Canada. This is a very important topic, but the discussion can be markedly improved. I do encourage a statistical review for the points I mention below. | Thank you for your comments! |
| Page 4: The sentence “Ontario's benchmark for Wait 2 for the least urgent category of most non-cancer conditions is within 184 days of deciding to proceed with surgery” is helpful. I was going to ask for the other benchmarks as well, but getting to the Outcomes/Exposures section those seem out of scope. So perhaps move this sentence to the latter section. Please also confirm that this are the current benchmark and whether the benchmark has changed over the course of the study period. | The benchmark of 184 days have been in place since 2008  Because readers of earlier drafts wanted the benchmark information when the concept of wait times was introduced in Study Setting, we included the following statement in that methods section:  Ontario's benchmark for Wait 2 for the least urgent category of most non-cancer conditions is within 184 days of deciding to proceed with surgery and remained constant throughout the study period.  To reinforce the stability of this benchmark during the study period, we will now also repeat a portion of this statement in the “Outcome and Exposure” section:  The benchmark for all these procedures of 184 days for Wait 2 did not change during the study period. |
| The paragraph in the discussion beginning with “Our analyses demonstrate that there is no such thing as a “wait list" for elective surgery” can improve with additional context. It makes it sound random; that a patients' wait-time depends on the surgeon and hospital. Rather, I don’t believe it’s random. Where the patient lives (e.g. urban vs rural; travel distance to nearest center; population density), who they (or their referring GP) knows, and competing demands on surgeon and hospital time matter, and are not random. | To better reflect the context we reworded the section as follows:  Our analyses demonstrate that there is no such thing as a “wait list” for elective surgery in Ontario; rather, every patient awaiting elective surgery is effectively in a unique queue, separate from others in the system, with each queue having a wide range of wait-time variations. A meaningful amount of the variation in wait times is attributable to hospitals, ranging from 6.2% for gallbladder surgery to 23.0% for cataract surgery. Surgeon-related variation ranged from 16.0% for non-cancer uterine surgery to 28.0% for cataract surgery. |
| The following paragraph states “our findings suggest there are efficiency gains to be made by improving coordination between the supply and demand of services”, but aside from demonstrating variation in wait-times by surgeon and hospital, this statement is not supported by the data. | We have rephrased this sentence: Our findings are hypothesis-generating, indicating that there might be efficiency gains from improving coordination between the supply and demand of services. |
| The statement “this analysis suggests that reporting mean or median wait times, or the proportion of patients not meeting waiting time targets, is an inadequate measure of system performance in access to care” is also not clearly evident from the results. Why are wait-times inadequate measures of system performance? No data are shown on variability in the proportion of patients receiving surgery within wait-time targets. There may indeed be very little variability on this measure (and therefore distributional efficiency). Does Ontario Health target wait-times or wait-time targets? | We have rephrased the sentence to clarify our intent:  Finally, given the wide variation in the surgical wait times of patients treated within the same hospital (and even by the same surgeon), the reporting of mean or median wait times—or the proportion of patients not meeting waiting time targets—may be an inadequate measure of overall system performance in access to care, since it obscures the true extent of wait time variation. |
| Again, the analysis did not suggest this: “However, our analysis suggests that increasing the supply of surgical services will not reduce the longest wait times without a coordinated system that better matches demand to supply.” The statement seems to imply that surgeons are standing around idle, when we know that’s not the case. It's an interesting discussion point, but it's not data-driven. | Good point. We have reworded the sentence as follows: However, given the significant variation in wait time between surgeons and hospitals, only increasing the supply of surgical services will not necessarily reduce the longest wait times without a coordinated system that better matches demand to supply. |
| Is a “Single-entry referral model” equivalent to a centralized referral system? This is an important paragraph. Some additional context about the existing referral system can help here. Are there any incentives to existing referral practices that may pose a barrier to a single-entry referral model? Do you think that busier GPs spend less time trying to get an earlier referral to a specialist? But all this discussion seems to hover around Wait 1. Once the decision to treat is made, what does a single-entry referral model offer? I presume the biggest barrier is OR time (and staff) | To clarify our argument, we edited the discussion here as follows:  A policy intervention promoted by many analysts and health system leaders that could reduce the extreme variation in wait times is the broader use of single-entry models and team-based care. [21–23] Single-entry models, also referred to as “central intake” models, are informed by queuing theory and can reduce excess variation in wait times for consultations, and also for surgery, by balancing clinical loads among surgeons and hospitals and smoothing out the mismatch between supply and demand. Single-entry referral models may improve system performance by reducing variation in wait time, improving allocative efficiency, and reducing inequity in access to timely surgical care. The principle underlying single entry models can also be applied to patients who have already been assessed by a surgeon and are awaiting their surgical procedure. Assignment to surgery by the next available surgeon within a hospital or geographic region in a “team-based” or “shared care” model, as compared with restricting patients to have surgery by the same surgeons who provided the initial consultation, can further reduce variations in Wait 2 times. |
| The conclusion mentions inequity, but there was no data on measures of inequity. The point about single-entry referral system is not a conclusion of the study, but a point for discussion and should be removed from the conclusion. | We have removed the point about inequity and a single entry referral system. |
